# Sentinel lymph node biopsy: technique validation at the Setúbal Medical Centre, Portugal

**DOI:** 10.3332/ecancer.2008.124

**Published:** 2009-01-15

**Authors:** P Ferreira, R Baía, A António, J Almeida, J Simões, JC Amaro, C Quintana, L Branco, MV Rigueira, M Gonçalves, EV Pereira, LM Ferreira

**Affiliations:** 1 Senology Unit, General Surgery Service, Setúbal Medical Centre, Portugal; 2 Senology Unit, Gynecology & Obstetrics Service, Setúbal Medical Centre, Portugal; 3 Pathology Service, Setúbal Medical Centre, Portugal

## Abstract

**Aims:**

To evaluate the accuracy of sentinel lymph node biopsy in breast cancer patients at this institution, using combined technetium-99m (^99m^Tc) sulphur colloid and patent blue vital dye.

**Methods:**

From March 2007 to July 2008, 50 patients with a tumour of less than 3 cm and with clinically negative axillary lymph nodes underwent sentinel lymph node biopsy (SLNB), followed by axillary lymph node dissection (ALND). Sub-areolar ^99m^Tc sulphur colloid injection was performed the day before surgery, and patent blue vital dye was also injected sub-areolarly at least 5 minutes before surgery. Sentinel lymph node was identified during the surgical procedure, using a gamma probe and direct vision. All sentinel nodes underwent frozen section analysis. Later haematoxylin and eosin staining and immunohistochemical analysis were performed. Finally, SLNB was compared with standard ALND for its ability to accurately reflect the final pathological status of the axillary nodes.

**Results:**

The sentinel lymph node (SLN) was identified in 48 of 50 patients (96%). The number of sentinel lymph nodes ranged from one to four (mean 1.48) and non-sentinel nodes ranged from seven to 27 (mean 14.33). Of the 48 patients with successfully identified SLNs, 29.17% (14/48) were histologically positive. Sensivity of the SLN to predict axilla was 93.75%; accuracy was 97.96%. The SLN was falsely negative in one patient—6.25% (1/16).

**Conclusions:**

The SLNB represents a major advance in the surgical treatment of breast cancer as a minimally invasive procedure predicting the axillary lymph node status. This validation study demonstrates the accuracy of the SLNB and its reasonable false negative rate when performed in our institute. It can now be used as the standard method of staging in patients with early breast cancer at this institution.

## Introduction

Breast cancer is the most common malignancy among women in Europe, accounting for 20% or more of all cancers and representing the leading cause of cancer deaths in females between 35 and 55 years old in Europe. About one in 12 will develop the disease before the age of 75 years, representing a lifetime risk around 8% [1,2]. It is important that effective screening methods and accurate ways for staging and prognosis once the diagnosis has been established are available [3,4]. Axillary lymph node dissection (ALND) provides information about disease stage, local control of disease, and helps in the decision making for adjuvant therapy [5–7]. However, for patients with pathologically negative lymph nodes survival rates are not increased by ALND [8], and there are a considerable number of related complications, such as sensory nerve damage, haemorrhaging, seroma formation (20–55% of cases) [9,10] and chronic lymphoedema of the arm (7–56%) [11,12].

As about 60–70% of patients with early breast cancer have no regional axillary lymph node metastasis [12], sentinel lymph node biopsy (SLNB) is an easy to establish, ideal alternative, capable of accurately predicting the state of axillary lymph nodes, avoiding classical axillary lymph node staging and its consequent morbidity.

After being first described by Cabanas ***et al*** in 1977 [50], for carcinoma of the penis, the SLNB technique was then used in staging malignant melanoma, as reported in 1992, by Morton ***et al*** [13], and more recently for breast carcinoma as reported by Krag ***et al*** in 1993 [14] and Giuliano ***et al*** in 1994 [15]. The SLNB serves as a stand alone method for determining axillary nodal status, providing physicians with the ability to distinguish positive axillary lymph nodes in a relatively simple, safe, rational and accurate fashion.

The sentinel lymph node (SLN) is the first lymph node to drain the entire lymphatics of the breast. Since metastatic breast cancer cells travel via this route, an SLN free of metastatic cancer predicts the status of the remaining axillary nodes as also negative for metastasis [16,17].

Over the past 14 years, sentinel node biopsy in breast cancer patients has become an exciting research topic. Many studies have shown that SLNB accurately predicts axillary lymph node status [18–20] and is associated with less morbidity than ALND completion [9,21,22]. Results from international breast cancer centres show that, with the use of optimal techniques, SLNB predicts axillary nodal status with high accuracy and low clinical false-negative rates [20,23–26]. Many medical centres adopted SLNB without completion of ALND in patients who have a clinical negative SLN, in an effort to decrease the morbidity of axillary lymphoadenectomy while maintaining accurate staging [27,28].

The purpose of this study was to evaluate the accuracy of SLNB at this institution and to thereafter implement it as the standard method of staging in patients with early breast cancer.

Using radiolabelled nanocolloid and patent blue vital dye as tracers, SLNB was performed in patients with breast cancer and its feasibility evaluated for patients in our institution.

## Patients

Fifty consecutive breast cancer patients, with a primary tumour less than 3 cm and a clinically negative axilla, were included in a prospective study performed between March 2007 and July 2008 at our hospital. Both mastectomy and breast conservation patients were equally eligible. All patients undergoing the study gave their informed consent for sub-areolar injection of radiotracer and patent blue vital dye and for the surgical procedure to be performed. The trial was approved by the institutional review board. The age of the patients ranged from 36 to 79 years, with a mean age of 59.4 years. All patients underwent SLNB and consecutive axillary dissection. Used inclusion and exclusion criteria are listed in Table 1.

## Methods

During study enrolment at our institution, the SLNB technique included the use of technetium-99m (^99m^Tc) sulphur colloid and blue dye. The ^99m^Tc sulphur colloid was prepared by the nuclear medicine departments of two other validated institutions with which our hospital has an agreement (**Hospital CUF Descobertas** and **NuclearMed—Instituto de Medicina Nuclear**), and comprised a solution containing 1.0mCi (1.0mCi = 37MBq) ^99m^Tc labelled rhenium sulphide colloid (nanocolloid particles < 80 nm—**Nanocis®**). A total volume of 0.4 ml was injected sub-areolarly 15–24 hours before the operation. Planar scans of the involved breast and axillary area, in anterior and lateral projections, were acquired 15–30 minutes and 3 hours after tracer injection, to ascertain the overall distribution of the radiotracer and identify SLN. A skin mark was made over the first spot to become hot to facilitate SLN location during the operation.

With the patient already anaesthesiated, and at least five minutes before surgery, blue dye (**Bleu Patente V Sodique Guerbet**^®^ 2.5%) was injected sub-areolarly in four deposits around the nipple totalling 2 ml. The patient was then prepped and draped in the usual sterile fashion. A low-transverse axillary incision was made, and a handheld gamma probe (**Neo2000^®^** Gamma Detection System, Neoprobe Corp., OH, USA) was used to guide the dissection for hot-spot detection. A hot spot was defined as the spot that had the greatest radioactivity counts in the lymphatic basin, which was at least 25 counts per ten seconds or greater. All ‘hot’ nodes and blue nodes were removed. After removing all nodes, the activity of the resection bed was assessed and should be less than 10% of the hottest, most radioactive excised lymph node. The SLN specimen was sent for frozen section analysis, and regardless of the result, all patients underwent ALND.

## Pathological examination

All blue and ‘hot’ SLN specimens were carefully identified and evaluated by a pathologist. All lymph nodes in the specimen were identified and dissected from the surrounding tissue by the pathologist. The number of nodes and their dimensions were accessed. SLNs smaller than 10 mm did not undergo frozen section analysis, SLNs ≤ 5 mm were processed and totally included in paraffin blocks (one block with all SLNs ≤ 5 mm), SLNs > 5 mm and < 10 mm were sectioned ± 2/3 mm perpendicularly to the longitudinal axis, processed and totally included in paraffin (one block per SLN > 5 and < 10 mm) and SLNs ≥ 10 mm underwent frozen section analysis. SLNs > 10 mm were longitudinally bisected and one of the half sections was submitted to frozen section analysis (one to three frozen sections per analysis) and/or cytological analysis (**imprint**). Those not subjected to frozen sectioning were then processed and totally included in paraffin (two blocks per SLN ≥ 10 mm). All SLNs were sectioned (100 μm sections) and every other section (first, third, fifth, seventh, etc.) stained with haematoxylin and eosin (H&E). The corresponding section (second, fourth, sixth, eight, etc.) was stained immunohistochemically, using a cytokeratin cocktail of monoclonal antibodies that recognize a wide range of high- and low-molecular weight keratin peptides (AE1/AE3, **Dako^®^**). Both SLNs and non-nodal tissue collected from ALND were sectioned and examined for the presence of tumour cells.

## Statistical analysis

Important data, including patient demographics and tumour characteristics, were recorded in a **Microsoft® Office Excel** spreadsheet.

Accuracy was defined as the percentage of patients in which the sentinel node status has accurately represented the lymph node status of patient. False negative (FN) rate was defined as the percentage of patients who had histologically negative SLNs but other positive axillary nodes. Fisher’s exact test and χ^2^ tests were used to analyse the impact of different factors on this method.

## Results

A total of 50 consenting patients were enrolled in the study. The clinical data of the patients and their disease characteristics are shown in Tables 2 and 3. All the 50 patients who underwent lymphoscintigraphy had lymphatic drainage towards the axilla.

Breast surgery consisted of mastectomy in 17 patients (34%) and breast conservative surgery in 33 patients (66%). The average primary tumour size in the study population was 1.9 cm, ranging from 0.6 to 4.0 cm, including 36 T1 tumours (72%) and 14 T2 tumours (28%). There was only one tumour of more than 3 cm after pathological examination. As for histological features, there were 42 infiltrating ductal cancers (84%), two infiltrating lobular cancers (4%), one mixed carcinoma (2%), two papillary invasive carcinomas (4%), one micropapillary invasive carcinoma (2%) and two mucinous carcinomas (4%).

The SLN was successfully detected in 48 of the 50 patients, corresponding to a surgical overall identification rate of 96% using both dye and radioisotope, not statistically different to other equivalent studies. The patients in which detection was not accomplished were 57 and 76 years old, with a tumour size of 15 and 16 mm, respectively, both in the lateral superior quadrant location.

In the 48 successful cases, the number of nodes detected ranged from one to four, and the mean number of sentinel lymph nodes per case was 1.48. All SLNs were located at level 1 of the axilla. The number of dissected non-sentinel lymph nodes ranged from seven to 25, with a total of 688 nodes and a mean of 14.33 nodes/case.

**Figure 1:**
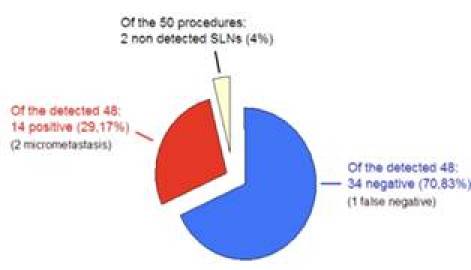
Summary of results.

A total of 14 patients (29.17%) had SLN positive for metastasis by definitive histological analysis, two of which were micro-metastasis (< 2 mm), not detected by frozen section analysis. Only 25% of cases with T1 lesions were found to have axillary lymph node metastasis compared to 42.86% of cases with T2 lesions. SLN analysis was negative in 34 patients (70.83%); however, metastatic cells were found in two lymph nodes of one patient after axillary dissection, accounting for the only FN result (FN rate 6.25% (1/16)), (Figure 1 and Table 4).

The FN finding occurred in a 79-year-old woman with a 28-mm ductal invasive carcinoma located in the medial superior quadrant of the breast.

These data give us a sensivity of 93.75%, a specificity of 100%, a positive predictive value of 100% and a negative predictive value of 97.06%, with an overall accuracy of 97.96% for predicting the malignant status of the axilla.

In five cases, the SLN was the only positive node, four of which had a ductal invasive tumour histological type and one had a micropapillary invasive pattern.

Our detection rate reached 100% after six SLNB procedures, and our FN occurred at the 15th procedure.

## Discussion

The SLN is the first lymph node to receive the lymphatic drainage of a metastatic tumour. In theory, there is a spread of tumour cells from sentinel nodes to other nodes. If so, further spread of cancer cells can be predicted by the SLNs [13,14]. The SLNB procedure in breast cancer patients is already a regular procedure in western countries.

The purpose of this study was to prospectively evaluate the accuracy of the SLNB in patients with early stage breast cancer undergoing this procedure by surgeons at this institution, with completion of ALND for confirmation.

Current literature frequently mentions that an identification rate greater than 90% and a FN rate of no more than 5–10% is a reasonable goal for surgeons and institutions developing the SLNB technique [29]. The accuracy of SLNs to predict axillary status should ideally be greater than 95%.

The identification rate in this study, using sub-areolar injections of dye/tracer, is 96%. The first study of Giuliano ***et al*** reported a 65% identification rate with blue dye [15], and their more recent studies achieve an identification rate of 93% and 99% of cases [30,31].

Our detection failures occurred in the first and sixth cases, gradually improving thereafter the dissection and identification of the SLN specimen. An identification rate of 100% in the later cases of our study reflects the plateau of the learning curve for this technique [32]. Some studies have indicated that the success rate is lower in patients older than 50 years [33], which is in accordance with our results.

Some reports suggest that the radiotracer and the blue dye are complementary, facilitating SLN detection when used together, thus accelerating the learning curve [34–36].

Despite controversy, we adopted the sub-areolar site for blue dye and radiotracer injection based on the hypothesis that breast drains lymph as a single unit, due to its embryological development from ectodermal primitive milk streak that becomes the areolar complex, there is also some evidence of high SLN identification rate and low FN rates and a rapid learning curve using this site of injection [37].

The FN rate is of great importance, especially as the true axillary nodal status has prognostic value and that this procedure is to be applied as a treatment protocol. In our study, the observed FN rate was 6.25%, not statistically different to other equivalent studies. Several series showed that FN SLNs occurred in tumours larger than 15 mm [34,38]. In this study, the false-negative SLN occurred in a 28-mm tumour.

In our study, the identification rate and FN rate are similar to other preliminary studies that used radiotracer and blue dye (Table 5).

Barnwell ***et al*** [40] assessed the success and accuracy of SLNB with blue dye and ^99m^Tc sulphur colloid compared to ALND. SLN was found in 38 of the 42 (90%) cases with no FNs. In a study by Nwriaku ***et al*** [18], using both blue dye and radiotracer, 119 women with breast carcinoma who underwent SLN and ALND, the SLN identification rate was 81% with one FN in a patient with axillary disease (FN rate of 4%). Mertz ***et al*** [42] analysed the relevance of the sub-areolar injection for SLN detection in multiple foci breast cancer, comparing 79 breast cancer patients who underwent ^99m^Tc filtered sulphur colloid sub-areolar injection (group I) with 32 patients who underwent peritumoural injection (group II). SLN was detected in 97.9% and 96.9% of the cases, respectively. No FNs were observed in group I and one FN in group II (FN rate 10%), which was related to a cancer with histological multiple invasive foci. Borgstein ***et al*** [43] performed periareolar injection of blue dye and peritumoural radioisotope in 130 patients and achieved a 96.9% identification rate and a 0% FN rate. Smith ***et al*** [44] compared 19 patients who received ^99m^Tc sub-areolar and peritumoural blue dye injections with 19 patients who received peritumoural injection of both blue dye and radiotracer. SLNs were found in all patients of the first group and in 18 patients of the second group. The FN rate was 0% for the first group and 20% for the second group. McMasters ***et al*** [36] documented a 98.8% identification rate and a 5.9% FN rate, using sub-areolar radiotracer injection and peritumoural blue dye injection in 85 patients. Donahue [45] obtained an identification rate of 100% and a FN rate of 8.3%, using peritumoural radioisotope and sub-areolar blue dye injection in 42 patients. Kern [46] reported an identification rate of 98.4%, a FN rate of 0% and an accuracy of 100% for predicting the malignant status of the axilla in a study with 187 patients, using sub-areolar injection dual-tracer technique. Chapgar ***et al*** [47], reported a multi-centre clinical trial on 3961 patients. For 1762 patients given radiotracer injection, identification rates of 91.1% for peritumoural injections, 99.3% for sub-areolar injections and 95.6% for periareolar injections were obtained, with FN rates between 8% and 9%. D’Eredita ***et al*** [37] achieved high SLN identification rates (94.2–100%) but with FN rates of 9% in their first study, but this dropped to 0% in later studies, showing clearly the importance an adequate learning period, also sustained by Cody ***et al*** [48].

Our results are also similar to reports based on larger sample series such as Cody’s [29] meta-analysis of 12 validation studies from 1993 to 1999 and ALMANAC Trialists Group’s [49] prospective multi-centre validation study performed to quantify identification and FN rates of SLNB and evaluate factors influencing them, both using radiotracer and blue dye (Table 6) [29,49].

All these studies demonstrate that SLNB can accurately predict the axillary lymph node status. The FN results suggest that a correct inspection of the axilla and removal of any nodes that appear abnormal is an important part of the SLN procedure.

## Conclusion

Sentinel lymph node biopsy is considered to be the gold-standard method in staging patients with early-stage breast cancer and a clinically negative axilla, providing important prognostic information to plan adjuvant treatment and avoiding the morbidity of invasive ALND.

The present study reflects our experience, indicating that the SLNB can be performed in an accurate, safe and reproducible manner in our hospital and is now ready to be adopted as the standard staging procedure for patients with early breast cancer.

## Figures and Tables

**Table 1:**
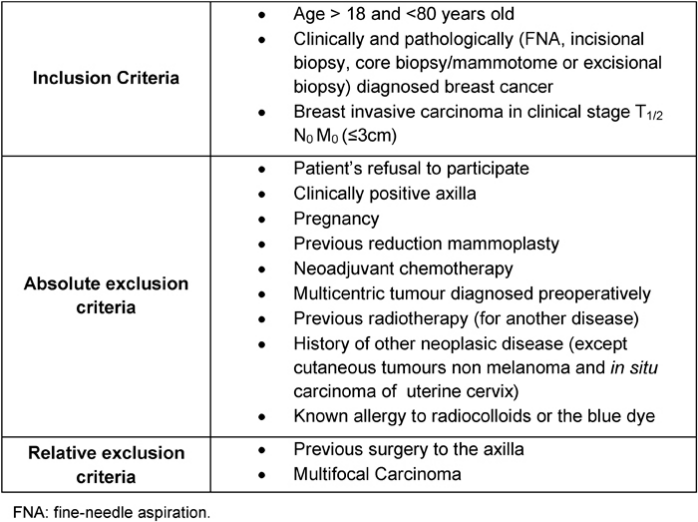
Inclusion and exclusion criteria

**Table 2:**
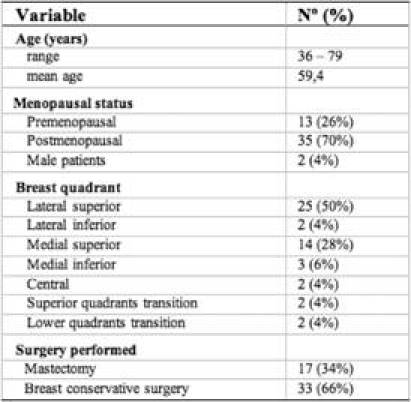
Patient clinical characteristics (*n*=50)

**Table 3:**
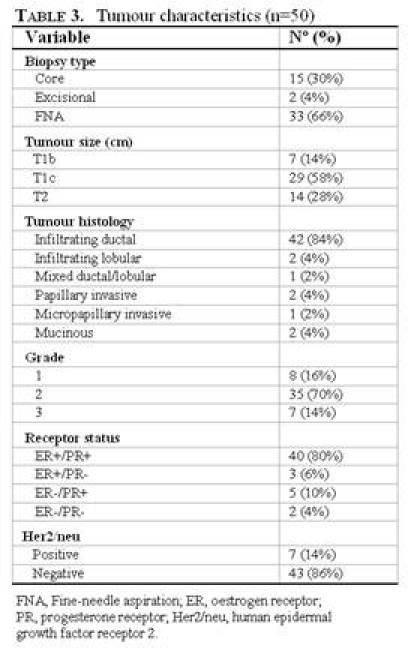
Disease characteristics (*n*=50)

**Table 4:**
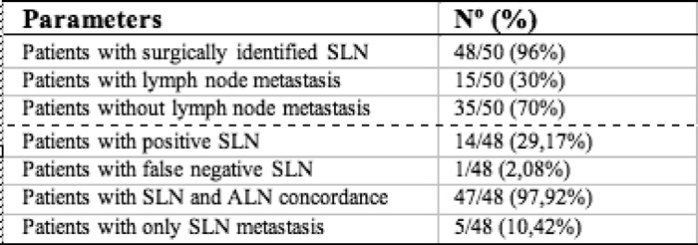
Summary of sentinel node biopsy

**Table 5:**
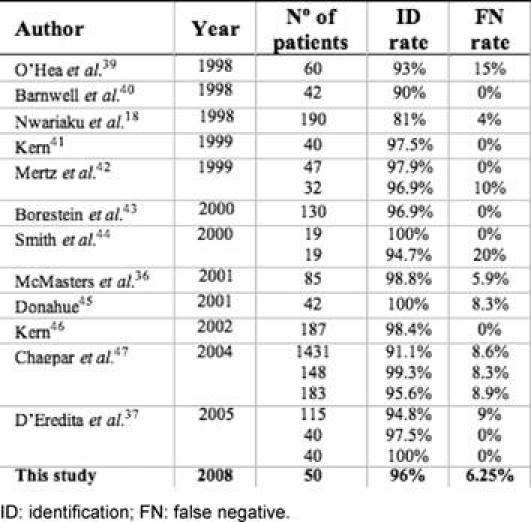
Identification and false-negative rates from similar sentinel lymph node biopsy studies

**Table 6:**
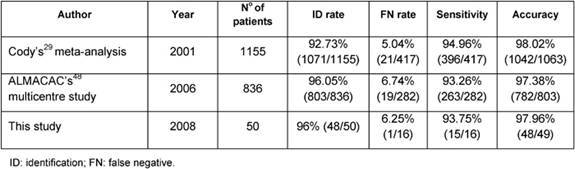
Comparison of this study with Cody’s meta-analysis and ALMANAC’s multi-centre study
